# The Field of Disease

**Published:** 1884-04

**Authors:** 


**The Field of Disease**: A book of Preventive Medicine. By B. W. RICHARDSON, M. D., LL. D., F. R. S., Fellow of the Royal College of Physicians, etc. Philadelphia: H. C. Lea's Son & Co. 1884.

The immense strides taken by medical science during the last quarter of a century have had no more conspicuous field of progress than the causation of disease. Not only has this led to marked advance in therapeutics, but it has given rise to a virtually new department of medicine—more important, perhaps, in its ultimate results than even the investigation of curative processes. Yet thus far there has been no attempt to gather into a systematic and intelligible shape the accumulation of knowledge acquired on this most interesting subject. Fortunately, the task has been at last undertaken by a writer who, of all, is perhaps best qualified for its performance, and the result of his labors can hardly fail to mark an epoch in the history of medical science.

The author states that he has written the book for those members of the intelligent reading public who, without desiring to touch the province of the physician and surgeon, wish to know the leading facts about the diseases of the human family, their causes and prevention. He has accomplished his purpose, for the work is written in a style and with a freedom from technicalities which makes it intelligible and interesting to the general reader. But while it is intended primarily for the public, we would also commend it very heartily to the profession; it contains much valuable information to the physician which it is difficult to find elsewhere and is worthy a place in every doctor's library.

